# The Absorbability of Fats or Analogous Substances by the Skin

**Published:** 1886-06

**Authors:** E.B. Angell

**Affiliations:** Rochester, N. Y.


### Translations.

### The Absorbability of Fats or Analogous Substances by the Skin.

Translated from the *Journal de Medicine de Paris*, March 7, 1886.

By E. B. Angell, M. D., Rochester, N. Y.

The celebrated dermatologist, Dr. Unna, has shown that the more readily a fatty substance absorbs water the more rapidly is it itself absorbed by the skin. He has found out what are the relative amounts of water that fatty substances will take up, such as vaseline, lanoline, and various mixtures. The complete table of the results of his experiments is given below. It is of interest through indicating the relative value of the various substances used as inunctions or as vehicles for external medication.

One hundred parts of the following substances absorb :

| | | | Parts of Water. |
|---|---|---|---|
| 1. | Vaseline, | | 4 |
| 2. | Lard, | | 15 |
| 3. | Benzoated Lard, | | 17 |
| 4. | Almond Oil, Yellow Wax, | 70<br>30 | 23 |
| 5. | Olive Oil, Yellow Wax (According to the age of the oil.) | 70<br>30 | 26 to 31 |
| 6. | Cod-Liver Oil, Yellow Wax, | 70<br>30 | 28 |
| 7. | Cod-Liver Oil, White Wax, | 70<br>30 | 32.3 |
| 8. | Linseed Oil, Yellow Wax, | 70<br>30 | 41.3 |
| 9. | Linseed Oil, White Wax, | 70<br>30 | 48.5 |
| 10. | Oleic Acid, Yellow Wax, | 70<br>30 | 50.5 |
| 11. | Oleic Acid, White Wax, | 70<br>30 | 60 |

| No. | Ingredients | Parts | Absorption |
|---|---|---|---|
| 12. | Olive Oil, 60; Turpentine (Oleo-resin), 10; Yellow Wax, 30 | 60 / 10 / 30 | 16 |
| 13. | Olive Oil, 65; Resin, 10; Yellow Wax, 25 | 65 / 10 / 25 | 19 |
| 14. | Mutton Tallow, 70; Olive Oil, 30 | 70 / 30 | 27 |
| 15. | Lard, 80; Spermaceti, 10; Olive Oil, 10 | 80 / 10 / 10 | 14 |
| 16. | Lard, 50; Spermaceti, 10; White Wax, 10; Olive Oil, 30 | 50 / 10 / 10 / 30 | 28 |
| 17. | Olive Oil, 70; Spermaceti, 15; Yellow Wax, 15 | 70 / 15 / 15 | 32.6 |
| 18. | Olive Oil, 70; Spermaceti, 15; White Wax, 15 | 70 / 15 / 15 | 39.5 |
| 19. | Lanoline | | 105 |

According to the above table, mixtures containing white wax absorb more water than those prepared with yellow or unbleached wax. This may be due to the fact that white wax is more or less acid, and this opinion seems to be confirmed by the greater absorbability of mixtures containing oleic acid.

